# Serious Illness Conversation–Evaluation Exercise: A Novel Assessment Tool for Residents Leading Serious Illness Conversations

**DOI:** 10.1089/pmr.2020.0086

**Published:** 2020-11-24

**Authors:** Jenny J. Ko, Mark S. Ballard, Tamara Shenkier, Jessica Simon, Amanda Roze des Ordons, Gillian Fyles, Shilo Lefresne, Philippa Hawley, Charlie Chen, Michael McKenzie, Isabella Ghement, Justin J. Sanders, Rachelle Bernacki, Scott Jones

**Affiliations:** ^1^ Department of Medical Oncology, University of British Columbia, BC Cancer—Abbotsford, Abbotsford, British Columbia, Canada.; ^2^ Department of Internal Medicine, Chilliwack General Hospital, Chilliwack, British Columbia, Canada.; ^3^ Department of Medical Oncology, BC Cancer—Vancouver, Vancouver, British Columbia, Canada.; ^4^ Department of Oncology, University of Calgary, Calgary, Alberta, Canada.; ^5^ BC Centre for Palliative Care, Vancouver, British Columbia, Canada.; ^6^ Department of Radiation Oncology, BC Cancer—Vancouver, Vancouver, British Columbia, Canada.; ^7^ Pain and Symptom Management/Palliative Care Program, BC Cancer—Vancouver, Vancouver, British Columbia, Canada.; ^8^ Ghement Statistics, Inc., Richmond, British Columbia, Canada.; ^9^ Ariadne Labs, Dana-Farber Cancer Institute, Boston, Massachusetts, USA.; ^10^ Vancouver Coastal Health, Vancouver, British Columbia, Canada.

**Keywords:** advance care planning, resident education, serious illness conversation

## Abstract

***Background/Objectives:*** The serious illness conversation (SIC) is an evidence-based framework for conversations with patients about a serious illness diagnosis. The objective of our study was to develop and validate a novel tool, the SIC-evaluation exercise (SIC-Ex), to facilitate assessment of resident-led conversations with oncology patients.

***Design:*** We developed the SIC-Ex based on SIC and on the Royal College of Canada Medical Oncology milestones. Seven resident trainees and 10 evaluators were recruited. Each trainee conducted an SIC with a patient, which was videotaped. The evaluators watched the videos and evaluated each trainee by using the novel SIC-Ex and the reference Calgary-Cambridge guide (CCG) at months zero and three. We used Kane's validity framework to assess validity.

***Results:*** Intra-class correlation using average SIC-Ex scores showed a moderate level of inter-evaluator agreement (range 0.523–0.822). Most evaluators rated a particular resident similar to the group average, except for one to two evaluator outliers in each domain. Test–retest reliability showed a moderate level of consistency among SIC-Ex scores at months zero and three. Global rating at zero and three months showed fair to good/very good inter-evaluator correlation. Pearson correlation coefficients comparing total SIC-Ex and CCG scores were high for most evaluators. Self-scores by trainees did not correlate well with scores by evaluators.

***Conclusions:*** SIC-Ex is the first assessment tool that provides evidence for incorporating the SIG guide framework for evaluation of resident competence. SIC-Ex is conceptually related to, but more specific than, CCG in evaluating serious illness conversation skills.

## Background

Advance care planning (ACP) is “a process that supports adults at any age or stage of health in understanding and sharing their personal values, life goals, and preferences regarding future medical care. The goal of ACP is to help ensure that people receive medical care that is consistent with their values, goals and preferences during serious and chronic illness.”^1^ Clinician-led ACP discussions about prognosis and patient preferences increase the quality of care perceived by patients and families and may reduce anxiety and depression.^2–6^

In patients with cancer, ACP conversations have been shown to be instrumental in both tailoring management plans that accord with patient values and communicating these plans with physicians and substitute decision makers.^7–10^ The ACP discussions should occur earlier in the cancer trajectory, but the majority still occur close to the end of life.^11–13^ Enhancing the skills of physician learners may provide a critical opportunity to improve the quality and timing of ACP.

Ariadne Labs developed the serious illness care program as a system-level intervention to improve the prevalence, timing, and quality of ACP in serious illness, through implementation of communication skills training and coaching and systems changes that support adoption and use of clinical conversation tools.^14^ The serious illness conversation guide (SICG), the signature clinical tool, comprises a seven-item framework to help clinicians share prognosis and elicit critical information to inform future decision making and care, including illness understanding, decision-making preferences, goals and fears, views on trade-offs and impaired function, and caregiver involvement.^15^ A number of health systems in which the current study was situated have adopted the SICG as the basic framework for ACP discussions in provincial cancer clinics.

Recently, the Royal College of Physicians and Surgeons of Canada has initiated restructuring of postgraduate medical training and assessment paradigms with a shift from a time-dependent to a competency-based model. A time-dependent model assumes acquisition of competency with a length of time in training, whereas competency-based medical education (CBME) focuses on outcomes and abilities as the organizing principle of curricular design. To our knowledge, no assessment tool has yet been developed or validated specifically for evaluation of trainee competency in SICs in oncology patients.

### Objectives

The objectives of our study were to develop and validate a novel tool, the SIC-evaluation exercise (SIC-Ex), for assessment of trainee competency in leading SICs with oncology patients in the ambulatory setting. This article presents results from the quantitative analyses; qualitative analyses from the narrative data have been presented as a conference abstract and will be reported in a separate article.^16^

## Methods

### SIC-Ex development

The study was approved by the provincial Research Ethics Board. We developed the SIC-Ex tool based on the mini-clinical evaluation exercise (CEx), the palliative care CEx, and SICG framework.^17–19^ The content and format of the checklist items were derived from and formatted based on the domains of conversations described in the SICG, and three steps that Sudore and Fried indicate as important for assessing patients' and surrogates' needs in preparing for in-the-moment decision making.^20,21^

This process has followed the guidelines for developing evaluation checklists published by the Joint Committee on Standards for Educational Evaluation.^22^ A draft of the SIC-Ex was constructed. This draft was reviewed for relevance, comprehensiveness, applicability, and clarity and it was finalized through an iterative process by palliative care physicians and content experts with an interest in medical education and SICs. Oncologists and program directors for academic oncology residency programs also reviewed the SIC-Ex for congruency with CBME models of assessment.

The final version of SIC-Ex covered four domains defined as trainee competency milestones, with specifically assigned questions to which each evaluator could give a numerical score between 1 (needs further instruction) and 4 (competent to perform independently) (Supplementary Appendix SA1):

- Communication basics (professional, communicator)^23^
-  ○ Demonstrated nonverbal empathy. For example, sat down, made eye contact
-  ○ Demonstrated verbal empathy. For example, named emotions, understood emotions, stated respect for patient, offered support
-  ○ Used open-ended questions
- Introducing ACP (professional, communicator, health advocate)
-  ○ Introduced ACP as a relevant topic for this patient, for example, benefit for patient/family, “Hope for the best, prepare for the worst”
-  ○ Clarified components of ACP previously engaged in
-  ○ Obtained permission from patient/family to proceed
- Learning about the patient (professional, communicator, leader, scholar)
-  ○ Understanding: clarified patient's understanding of illness (including diagnosis, treatments, prognosis)
-  ○ Information preferences: assessed patient readiness to engage in ACP conversation. For example, some patients like to know about time, other like to know what to expect, others like to know both, others neither.
-  ○ Prognosis: shared prognosis of current illness with patient, tailored to information preferences
-  ○ Goals: inquired about patient's own values and health care goals if medical condition worsens.
-  ○ Fears/worries: explored patient's fears and/or worries with regard to the future of his/her health.
-  ○ Function: explored activities that the patient deems critical to having an acceptable quality of life.
-  ○ Trade-offs: explored medical treatments the patient would be willing to go through to gain more time living.
-  ○ Family.
-   ➢ Explored how much the patient's family/friends may know about his/her priorities and wishes
-   ➢ Determined whether there were other important friends or family members who needed to be included in future ACP conversations
-   ➢ Asked who the patient would like as a substitute decision maker
- Planning (professional, communicator, leader, collaborator)
-  ○ Affirmed commitment to continue caring for patient
-   ➢ Acknowledged medical realities
-   ➢ Summarized key goals/priorities
-   ➢ Described treatment options that reflect goals/priorities
- Made recommendations about the next steps
- Documented conversation
- Provided patient with written information pertaining to local ACP policies (e.g., ACP conversation guide)

It also included an ordinal global rating (“Overall what is your impression about how this student led the ACP conversation?”) capturing the evaluator's impression of the trainee's overall competence (scores 1–4, where 1 = very satisfied and 4 = very dissatisfied).

### SIC-Ex validation

We recruited volunteer trainees and preceptors (evaluators below) from three academic cancer centers. Volunteer patients were recruited from three cancer clinics. All volunteers provided informed consent. Volunteer trainees received a 15-minute introduction to the SICG, but they did not receive standardized training. Each trainee was assigned to a volunteer patient and asked to lead a single SIC in an outpatient oncology clinic. We videotaped each session. Trainees completed a self-assessment survey immediately after the SIC.

After watching the videotapes, evaluators used the SIC-Ex tool (experimental tool) and Calgary-Cambridge guide (CCG; reference tool) to score trainee performance at month 0 (defined as the first time that the evaluator assessed the scores within six months of completing the discussion). The CCG was chosen as a reference tool as it is a validated, widely used, and well-known evaluation tool for trainees to evaluate their communication skills, with relevant domains comparable to the domains in the SIC-Ex—Initiating the session; Gathering information; Providing structure; Building relationship; Explanation and planning; Closing the session; Options in explanation and planning.

The same evaluators watched the videos again three months later and rated them by using the same tools. This article presents results from the quantitative analyses; qualitative analyses from the narrative data have been presented as an abstract and will be reported in a separate article.^16^ All statistical analyses were performed by using the open source software R.^24^ We adopted Kane's framework^25,26^ to generate validity evidence for the SIC-Ex as follows:

(1) Defining the proposed use: SIC may occur within a single discussion or over several discussions at different times and with different health care professionals. For the purpose of the study, all of the domains of the SIC-Ex were evaluated to standardize tool administration. The tool is intended for oncology trainees at all levels of training (e.g., residents, fellows).
(2) Scoring: We defined it as transformation of observation(s) into an insightful and accurate response in the forms of scores and narratives.
(a) Quantitative assessment: The SIC-Ex was designed to generate both a global ordinal score and a composite of scores in different domains of competence in serious illness (SI) discussion. The tool does not have pass/fail cutoffs; rather, the tool utilized a Likert scale (scores 1–4) to describe competence in each domain, and a global impression of overall competence (scores 1–4). The total score across each domain as well as across all four domains was calculated to obtain a numeric score describing overall competence for the analysis. The scores were meant to be utilized as points for discussion and feedback in a similar fashion as the mini-CEx.
(b) Qualitative assessment: An open-ended question at the end of the SIC-Ex captured narrative feedback from the evaluators to add richness, authenticity, and clarification of the quantitative data. These data are not discussed in this article and will be described in a separate article.
(3) Generalization (Synthesis of individual data elements into an insightful and accurate overall interpretation regarding performance in the test setting)
(a) Item analysis: The distributions of item-specific scores on SIC-Ex and CCG scores were summarized via their median and range.
(b) Inter-evaluator reliability: We used average score of intra-class correlation coefficients (ICCs) for the (quantitative) domain-specific and total scores across domains on SIC-Ex at month zero. We used Brennan-Predigger's and Gwet's AC1 coefficients and associated 95% confidence intervals (CIs) for the (ordinal) global rating on SIC-Ex at months 0; both types of coefficients were categorized by using Altman's benchmark scale as Poor (<0.2), Fair (0.2–0.4), Moderate (0.4–0.6), Good (0.6–0.8), or Very Good (0.8–1).^27^
(c) Evaluator agreement with group average: We used modified Bland-Altman plots, which account for more than two evaluators to assess agreement between individual evaluators and the group average in terms of (quantitative) domain-specific and total scores across domains on SIC-Ex at month 0.^28^
(d) Intra-evaluator reliability: Intra-evaluator (or test-retest) reliability of (quantitative) domain-specific and total scores across domains on SIC-Ex were computed by replacing the evaluator, trainee, interaction, and error variance components with their estimated values in the formula:

$$\frac{\sigma_{evaluator}^{2}+\sigma_{trainee}^{2}+\sigma_{evaluator:trainee}^{2}}{\sigma_{evaluator}^{2}+\sigma_{trainee}^{2}+\sigma_{evaluator:trainee}^{2}+\sigma_{error}^{2}},$$

where the variance components correspond to a linear mixed-effects model that treats each type of evaluator score as the outcome variable (measured at month zero and three for each evaluator-trainee combination), while including a fixed-effect intercept and random effects for the evaluator, trainee, and their interaction.

Intra-evaluator reliability of the (ordinal) global rating on SIC-Ex at month zero was calculated separately for each evaluator via Gwet's AC1 and Brennan-Prediggers' reliability coefficients, then summarized across evaluators, and finally interpreted based on Altman's benchmark scale.^27^

(4) Extrapolation (Extension of generalized interpretations from the test setting) into real-life situations (i.e., extrapolation beyond the evidence into a new context))
(a) Correlation with CCG: For each evaluator, the correlation between the (quantitative) total scores across all domains of the SIC-Ex and CCG instruments was computed at months zero by performing simple linear regressions involving the two sets of scores and computing Pearson's correlation coefficients; correlation coefficients of 0.4 or higher and ideally larger than 0.6 were desired.
(b) Agreement between trainees and evaluators: Agreement at month zero between trainees and evaluators was assessed by adding the trainee total self-scores on SIC-Ex to the evaluator total scores—separately for each domain and for all domains combined—and computing Krippendorff's *α*. The magnitude of *α* was interpreted in relation to 0 (perfect disagreement) and 1 (perfect agreement). Further, a decrease in the value of *α* compared with the one obtained by using only the evaluator ratings was interpreted to indicate that the added self-ratings did not influence inter-evaluator agreement negatively, implying that evaluators agree with trainees.^27^
(5) Implications (Translation of assessment results into meaningful decisions and actions, and the downstream effects of such decisions)

The global ordinal rating and domain scores from the SIC-Ex were provided to each of the trainee participants, along with the narrative comments provided by evaluators. Trainees had a de-briefing session with their preceptors after the video recording and completed the Student Post-Encounter Probe (Supplementary Appendix SA2) to determine their perception of: time/difficulty involved in completing the exercise, self-perceived competence in conducting elements of or the whole discussion under observation, and the educational value of the SIC-Ex. The preceptor also recorded his or her own response to two post-intervention questions that asked about the utility and process of the SIC-Ex (see Supplementary Appendix SA2).

## Results

We recruited seven residents and seven patients from two study sites (Abbotsford, Vancouver) at the end of a one-year study period. Ten evaluators from three study sites (Abbotsford, Vancouver, Calgary) completed the first round of resident evaluations by using the SIC-Ex and Cambridge tools at month 0; nine of these completed the second round of resident evaluations at month three. To prevent potential conflict of interest, the three evaluators who recruited students did not evaluate the students they recruited; subsequently, their evaluations were removed from the final statistical analysis. All seven residents provided self-ratings on the SIC-Ex tool immediately after the conversation.

### Item analysis

The median score across trainees per item for SIC-Ex was 3.06 (range 2.54–3.32), and for CCG, 3.51 (range 2.73–3.77). The median scores on SIC-Ex were numerically lower than the median scores on Cambridge.

### Inter-evaluator reliability of quantitative scores generated by SIC-Ex (i.e., “does one evaluator rate similarly to group average for each domain for the same trainee?”)

For the quantitative scores generated by SIC-Ex (i.e., domain-specific scores and total scores across domains), the inter-evaluator reliability at month zero was evaluated by computing intra-class correlation coefficients (ICCs) based on the average of these scores across evaluators for each trainee. The results revealed moderate inter-evaluator reliability at month 0, whether we measured absolute agreement or consistency. Sensitivity analyses excluding outliers did not significantly impact the results.

### Inter-evaluator reliability of global ordinal rating generated by SIC-Ex (i.e., “does one evaluator rate similarly to group average for the global rating for the same trainee?”)

**Fig. 1.**
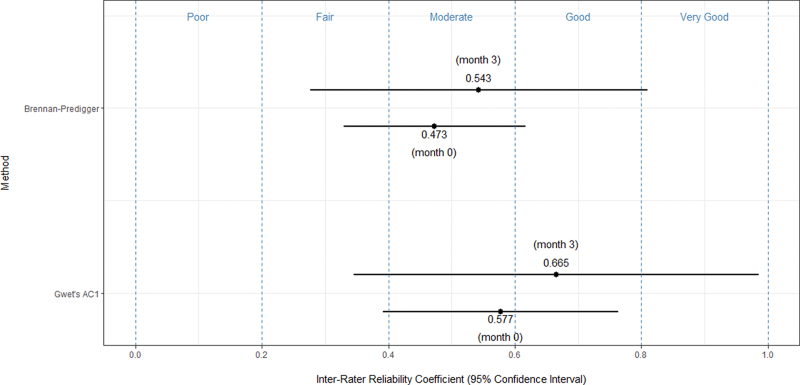
Inter-evaluator reliability at months zero for the (ordinal) global rating generated by SIC-Ex. SIC-Ex, serious illness conversation**–**evaluation exercise.

For the ordinal global rating generated by SIC-Ex, inter-evaluator reliability at month zero was assessed by computing Brennan-Predigger and Gwet's AC1 reliability coefficients and associated 95% CIs (Fig. 1). Both tests were performed and coefficients were computed to ensure that the results were comparable. The true inter-evaluator reliability was estimated as fair to good at month zero.

### Evaluator agreement with group average based on quantitative scores generated by SIC-Ex

**Fig. 2.**
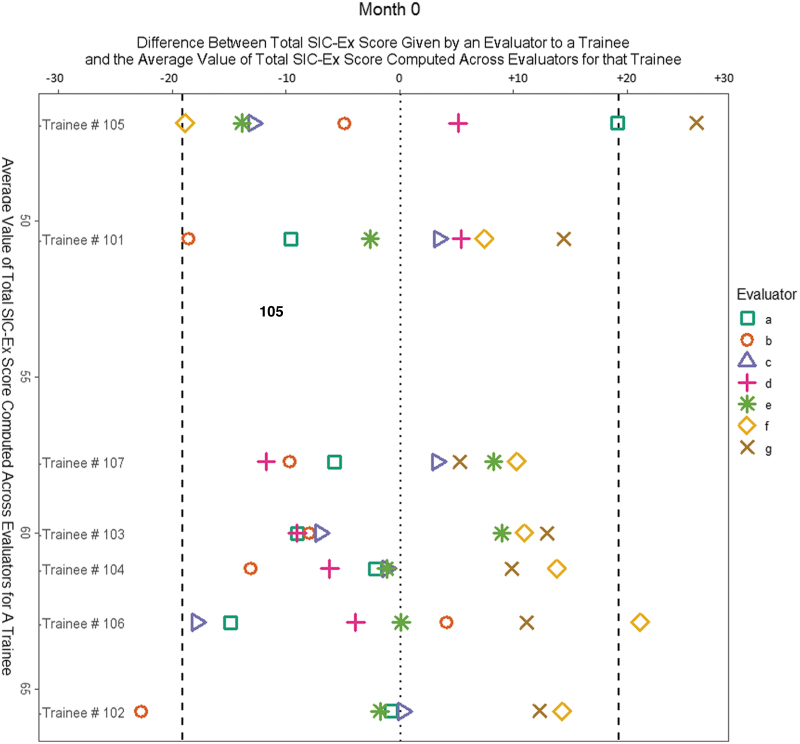
Modified Bland-Altman plots for the evaluators a–g, derived from the total domain scores across domains on SIC-Ex they assigned to the seven trainees at month zero. If the difference between the total SIC-Ex score given by a particular evaluator to a trainee and the average value of total SIC-Ex score computed across evaluators for that trainee was higher than or lower than ±20 (bold dotted vertical lines), the evaluators were considered outliers from the group average. Total SIC-Ex score refers to the total score across all domains of SIC-Ex.

Figure 2 describes the modified Bland-Altman agreement analysis of total scores across domains on SIC-Ex at month zero and shows that most evaluators scored a particular trainee similarly to the group average, except for one or two evaluator outliers who rated the same trainee significantly higher or lower than the group average. Analogous analyses for domain-specific scores on SIC-Ex at month zero revealed comparable findings (not shown).

### Intra-evaluator reliability of quantitative and qualitative scores generated by SIC-Ex (i.e., does an evaluator rate similarly to oneself for the same trainee between two timepoints?)

Intra-evaluator (test-retest) reliability of domain-specific and total scores across domains on SIC-Ex showed a moderate level of self-consistency of evaluators over time (intra-evaluator reliability ranged from 0.477 to 0.763 for domain-specific scores and was equal to 0.795 for the total score across domains) (Table 1). Intra-evaluator reliability of the (ordinal) global rating on SIC-Ex exhibited a “fair to good” level across evaluators (Table 2).

**Table 1.** Average Score Intra-class Correlation Coefficient Estimates and 95% Confidence Intervals for Specific Domains of Serious Illness Conversation–Evaluation Exercise and for All Domains Combined, Computed at Month Zero

Domain	ICC estimate at month 0 (95% confidence interval)	
	Agreement (n = 7)	Consistency (n = 7)
“Communication basics”	0.822 (0.531 to 0.964)	0.856 (0.599 to 0.971)
“Introducing ACP”	0.523 (−0.017 to 0.888)	0.666 (0.070 to 0.934)
“Learning about the patient”	0.526 (−0.069 to 0.894)	0.618 (−0.065 to 0.924)
“Planning”	0.596 (0.042 to 0.912)	0.669 (0.078 to 0.934)
All domains combined	0.605 (0.083 to 0.913)	0.702 (0.171 to 0.941)

ACP, advance care planning; ICC, intraclass correlation coefficient.

**Table 2.** Intra-Evaluator Reliability of Domain-Specific and Total Scores Across Domains Generated by Serious Illness Conversation–Evaluation Exercise

Domain	Intra-evaluator reliability at month 0 (?)
Communication basics	0.477
Introducing ACP	0.661
Learning about the patient	0.763
Planning	0.692
All domains combined	0.795

### Correlation with CCG (i.e., does SIC-Ex rate a trainee similarly as well as CCG?)

**Fig. 3.**
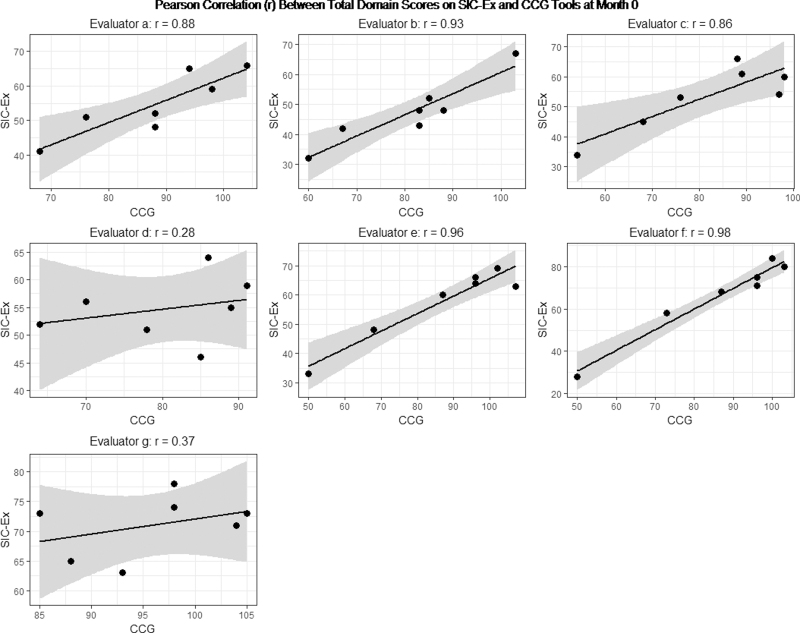
Correlation between total domain-specific scores and total scores across domains on the SIC-Ex and CCG tools at month zero. CCG, Calgary-Cambridge guide.

For most evaluators, total scores across domains produced by the two tools, SIC-Ex and CCG, were highly correlated at month 0 (Fig. 3). The two outlying evaluators d and g with low correlations of 0.28 and 0.37, respectively, at month zero were also outliers in regards to the inter-evaluator reliability at month 0.

### Agreement between trainees and evaluators based on quantitative scores generated by SIC-Ex (i.e., do trainees' self-scores on SIC-Ex match with those of the evaluators?)

The addition of trainee self-scores to the evaluator scores when computing Krippendorff's *α* at month zero did not influence inter-evaluator agreement negatively for the “Introducing ACP,” “Learning about the patient,” “Planning” domains, and for all domains combined, but it did negatively affect the “Communications basics” domain (Table 3). Although the agreement between trainees and evaluators was similar in magnitude to that between evaluators alone, it was on the low or very low side (Table 4).

**Table 3.** Intra-Evaluator Reliability of Ordinal Global Rating Generated by Serious Illness Conversation–Evaluation Exercise, Summarized across Evaluators

Intra-evaluator reliability coefficient	Average across evaluators	Standard deviation across evaluators	Range across evaluators (minimum to maximum)
Gwet's AC1	0.762	0.138	0.586–0.906
Brennan-Predigger	0.697	0.146	0.486–0.871

**Table 4.** Krippendorff's *α* Values at Month Zero Derived from (a) Evaluator Scores on Serious Illness Conversation–Evaluation Exercise (SIC-Ex) and (b) Evaluator Scores Plus Trainee Self-Scores on SIC-Ex. Both Domain-Specific Scores and Scores Across All Domains Were Considered for Trainees and Evaluators

Domain	Krippendorff's α at month 0	
	(A) Evaluator scores on SIC-EX	(B) Evaluator scores plus trainee self-scores on SIC-EX
Communication basics	0.323	0.304
Introducing ACP	0.0895	0.103
Learning about the patient	0.102	0.102
Planning	0.0559	0.0854
All domains combined	0.135	0.141

SIC-Ex, serious illness conversation–evaluation exercise.

## Discussion

To the authors' knowledge, our study is the first to demonstrate validity evidence of using SIC-Ex for resident trainees interviewing outpatient cancer patients. It brings together the Serious Illness Conversation framework in the context of competency-based medical education (CBME) evaluation and to validate domain-specific, total domain, and global scores and narrative comments. CBME is “an approach to preparing physicians for practice that is fundamentally oriented to graduate outcome abilities and organized around competencies derived from an analysis of societal and patient needs.”^29,30^ The Medical Oncology Subspecialty at the Royal College of Physicians and Surgeons of Canada defines “discussing serious news” and “transitioning away from active anti-cancer therapy” as professional activities that can be entrusted to trainees once their component core competencies have been achieved. The SIC-Ex was constructed around these competencies. As SIC-Ex is a performance-based, formative evaluation process composed of multiple key milestones and integrating multiple domains of competencies, the outcomes (competencies) are not isolated elements of knowledge or a skill, but rather are integrated and observed/measured to ensure their acquisition. Our study demonstrates that it is feasible to assess a trainee's competence by incorporating elements of a pre-existing evidence-based communication tool.

At least three studies have quantified and scored trainees' communication skills in end-of-life discussions or ACP by using non-validated internal scales, although these were based on self-assessment, which is generally a less accurate method of competency assessment, rather than preceptor observations.^31–33^ Previously validated assessment tools in medical communication skills include the CCG and mini-CEx^18,19,34^; however, they are generic tools for assessment of communication skills and are not validated to assess ACP conversation skills. Han et al. previously reported the feasibility and potential effectiveness of the palliative care CEx, a modified version of CEx, to assess trainees' ability to discuss end-of-life issues with patients; again, this tool is not specifically designed to assess ACP conversation skills.^17^ Another recent study developed and validated an instrument, ACP-Communication Assessment Tool (CAT), to assess ACP communication skills of clinicians and trainees.^35^ The ACP-CAT has domains that overlap with the SIC framework; however, the participating trainees did not have formal orientation to the SIC, and the conversations were done in the context of an examination with a simulated patient. Our study allows an incorporation of an evaluation instrument for SIC into the CBME for assessment of trainees in a real-world outpatient clinic setting.

Overall, our study demonstrated that the SIC-Ex scores for most of the domains were lower with more discriminatory variations among residents than those of the CCG for each resident. This may indicate an increased specificity of SIC-ex scores for the particular domains assessed by each question compared with more generic communications assessed by CCG. Literature suggests that generic evaluation scores for communication skills are not necessarily formative, as they may provide feedback that is often too general and not specific to the skillsets required for a certain milestone.^36,37^ Generic evaluation scores may not generate recommendations on how a learner can improve and build on the specific skill sets. Studies also suggest that generic evaluation scores may need to be tailored to the specific student group.^38^ This same evidence suggests that more tailored evaluation scores tend to improve the training group performance over time. In addition, the narrative data generated from the written comments by the evaluators are critical to the formative nature of the evaluation; this has been presented as a conference abstract^16^ and will be explored in a separate article. The SIC-Ex allows for the written comments in each domain and also for a global summary.

We observed overall high inter- and intra-evaluator variabilities. Evaluators had no formal training and did not undertake normative processes for evaluating residents using the SIC-Ex and CCG. Training and orientation on the use of SIC may reduce inter- and intra-evaluator variability. Although all of our evaluators were familiar with SIC, some may have been more comfortable with its use than others given their practice patterns. Another aspect is familiarity with the formative evaluation process itself. There are data to suggest that faculty development on use of evaluative tools and feedback mechanisms is essential to training evaluators to implement a successful mini-CEx assessment program.^39,40^ Guidance for evaluators on the CEx as a tool to foster the preceptor – student relationship may improve the effectiveness of the SIC-Ex process.

Trainee self-scores did not correlate well with evaluator scores. Other studies show poor correlation between trainees' self-assessment and preceptor, patient, or family reviews of a trainee's performance, particularly in communication competencies and outcomes of end-of-life care.^41–43^ Literature suggests that experiential training as well as a validated evaluative tool for formative feedback may lessen the gap between the self-assessment of trainees and evaluator/patient/family assessments.^43^ Given the importance of serious illness discussions in patients with cancer, a tool such as the SIC-Ex could help with a formative educational process in this area.

Our study shows that SIC-Ex can be used for the following purposes: (1) ongoing assessments of resident trainees in the outpatient oncology clinic settings to evaluate the CBME-based milestones, with comprehensive periodic reviews to ensure continued progress, as a trainee may receive both quantitative and qualitative feedback over a period of time to document progress in each domain and globally. However, we acknowledge that in this study, the residents were only assessed at one time point with no reassessment; (2) use of multiple assessors and assessments to enable the right assessment to be made at the right time for the right purpose, as the behavioral attributes evaluated through SIC-Ex are comprehensive and incorporate both generic attributes and those specific to serious illness conversations; (3) mechanisms to synthesize data collected through group processes to reach judgments about competence (e.g., as an Entrustable Physician Activity), as inter-evaluator reliability, evaluator agreement with the group mean, and intra-evaluator reliability are at least fair to good; (4) faculty development for all assessors, as it is critical for the evaluators to be familiar with the SICG; and (5) optimized relationships between the givers and receivers of formative feedback to enhance the incorporation of feedback into practice.^44^

The limitations of our study include the following: Due to small sample size, the generalizability of the study findings needs to be tested in a broader context. The current study did not test whether the SIC-Ex scores are responsive and sensitive to changes in the trainees' SIC skills. This will need to be examined in larger future studies. The discrepancy between trainees' self-scores and scores by evaluators (preceptors) may reflect a need to provide additional learner-centered instructions on use and evaluation of SIC to evaluators and trainees. The findings need to be taken in the context of small sample size and limited evaluator and/or trainee training on SIC before the study. Despite the validity evidence supporting use of SIC-Ex, the core Medical Oncology competencies were not developed around the SIC framework and therefore a broader use of SIC in oncology residencies needs to be examined in the current context of predefined milestones. In our statistical analysis, we used unweighted sums to compute the domain-specific scores and the total scores across all four domains of SIC-Ex. This assumes that all items of a domain are equally important in the determination of a domain-specific score, and that all items of SIC-Ex are equally important in the determination of the total score across domains. Due to the small sample size, and the fact that the items are extracted directly from the already established SIC framework, we did not further test each item or domain against the others in this study. Lastly, there may be possible selection bias in using tools on volunteer patients and volunteer learners.

In conclusion, this study adds validity evidence to support use of SIC-Ex in assessing resident competencies in serious illness communication. Use of the SIC-Ex has the potential to enhance formative evaluation and feedback process for residents having serious illness conversation with oncology patients. Further resident and evaluator training on SIC-Ex may enhance its reliability in evaluating domains that are specific to serious illness conversations.

## Supplementary Material

Supplemental data

Supplemental data

## Supplementary Material

Supplementary Appendix SA1

Supplementary Appendix SA2

## Abbreviations Used

ACP: advance care planning
CAT: Communication Assessment Tool
CBME: competency-based medical education
CCG: Calgary-Cambridge guide
CEx: clinical evaluation exercise
ICC: intraclass correlation coefficient
SIC: serious illness conversation
SIC-Ex: SIC-evaluation exercise
SICG: serious illness conversation guide
