# Supplementary material for: Serious Illness Conversation–Evaluation Exercise: A Novel Assessment Tool for Residents Leading Serious Illness Conversations
Source: Palliat Med Rep. 2020 Nov 24;1(1):280–90. doi: 10.1089/pmr.2020.0086 (PMC8241377; doi:10.1089/pmr.2020.0086)
Supplement: Supplemental data [file Supp_AppS1.docx]

**Serious Illness Conversation-Evaluation Exercise (SIC-Ex)**

Trainee #

Preceptor #

Patient #

EPA/IM stage

Date of discussion

Centre

To be filled out by the evaluating preceptor(s) watching the videotaped session

**Milestones (CanMEDS Roles):**

**Communication basics (Professional, Communicator)**

|  | 1 | 2 | 3 | 4 | N/A |
| --- | --- | --- | --- | --- | --- |
| Demonstrated nonverbal empathy  E.g. Sat down, made eye contact | Needs further instruction | Competent to perform with close supervision | Competent to perform with minimal supervision | Competent to perform independently | Not applicable |
| Demonstrated verbal empathy  E.g. Named emotions, understood emotions, stated respect for patient, offered support | Needs further instruction | Competent to perform with close supervision | Competent to perform with minimal supervision | Competent to perform independently | Not applicable |
| Used open-ended questions | Need further instruction | Competent to perform with close supervision | Competent to perform with minimal supervision | Competent to perform independently | Not applicable |

**Introducing ACP (Professional, Communicator, Health Advocator)**

|  | 1 | 2 | 3 | 4 | N/A |
| --- | --- | --- | --- | --- | --- |
| Introduced ACP as a relevant topic for this patient, e.g. benefit for patient/family, “Hope for the best, prepare for the worst” | Needs further instruction | Competent to perform with close supervision | Competent to perform with minimal supervision | Competent to perform independently | Not applicable |
| Clarified components of ACP previously engaged in | Needs further instruction | Competent to perform with close supervision | Competent to perform with minimal supervision | Competent to perform independently | Not applicable |
| Obtained permission from patient/family to proceed | Needs further instruction | Competent to perform with close supervision | Competent to perform with minimal supervision | Competent to perform independently | Not applicable |

**Learning about the patient (Professional, Communicator, Leader, Scholar)**

|  | 1 | | 2 | 3 | | 4 | N/A |
| --- | --- | --- | --- | --- | --- | --- | --- |
| **Understanding** | | | | | | | |
| Clarified patient’s understanding of illness (including diagnosis, treatments, prognosis) | | Needs further instruction | Competent to perform with close supervision | Competent to perform with minimal supervision | Competent to perform independently | | Not applicable |
| **Information preferences** | | | | | | | |
| Assessed patient readiness to engage in ACP conversation. E.g. some patients like to know about time, other like to know what to expect, others like to know both, others neither. | | Needs further instruction | Competent to perform with close supervision | Competent to perform with minimal supervision | Competent to perform independently | | Not applicable |
| **Prognosis** | | | | | | | |
| Shared prognosis of current illness with patient, tailored to information preferences | | Needs further instruction | Competent to perform with close supervision | Competent to perform with minimal supervision | Competent to perform independently | | Not applicable |
| **Goals** | | | | | | | |
| Inquired about patient’s own values and healthcare goals if medical condition worsens. | | Needs further instruction | Competent to perform with close supervision | Competent to perform with minimal supervision | Competent to perform independently | | Not applicable |
| **Fears/worries** | | | | | | | |
| Explored patient’s fears and/or worries with regard to the future of his/her health. | | Needs further instruction | Competent to perform with close supervision | Competent to perform with minimal supervision | Competent to perform independently | | Not applicable |
| **Function** | | | | | | | |
| Explored activities that the patient deems critical to having an acceptable quality of life. | | Needs further instruction | Competent to perform with close supervision | Competent to perform with minimal supervision | Competent to perform independently | | Not applicable |
| **Trade-offs** | | | | | | | |
| Explored medical treatments the patient would be willing to go through to gain more time living. | | Needs further instruction | Competent to perform with close supervision | Competent to perform with minimal supervision | Competent to perform independently | | Not applicable |
| **Family** | | | | | | | |
| Explored how much the patient’s family/friends may know about his/her priorities and wishes | | Needs further instruction | Competent to perform with close supervision | Competent to perform with minimal supervision | | Competent to perform independently | Not applicable |
| Determined if there were other important friends or family members that needed to be included in future ACP conversations | | Needs further instruction | Competent to perform with close supervision | Competent to perform with minimal supervision | | Competent to perform independently | Not applicable |
| Asked who the patient would like as a substitute decision maker | Needs further instruction | | Competent to perform with close supervision | Competent to perform with minimal supervision | | Competent to perform independently | Not applicable |

**Planning (Professional, Communicator, Leader, Collaborator)**

|  | 1 | 2 | 3 | 4 | N/A |
| --- | --- | --- | --- | --- | --- |
| Affirmed commitment to continue caring for patient | Needs further instruction | Competent to perform with close supervision | Competent to perform with minimal supervision | Competent to perform independently | Not applicable |
| Acknowledged medical realities | Needs further instruction | Competent to perform with close supervision | Competent to perform with minimal supervision | Competent to perform independently | Not applicable |
| Summarized key goals/priorities | Needs further instruction | Competent to perform with close supervision | Competent to perform with minimal supervision | Competent to perform independently | Not applicable |
| Described treatment options that reflect goals/priorities | Needs further instruction | Competent to perform with close supervision | Competent to perform with minimal supervision | Competent to perform independently | Not applicable |
| Made recommendations about the next steps | Needs further instruction | Competent to perform with close supervision | Competent to perform with minimal supervision | Competent to perform independently | Not applicable |
| Documented conversation | Needs further instruction | Competent to perform with close supervision | Competent to perform with minimal supervision | Competent to perform independently | Not applicable |
| Provided patient with written information pertaining to local ACP policies (e.g. ACP conversation guide) | Needs further instruction | Competent to perform with close supervision | Competent to perform with minimal supervision | Competent to perform independently | Not applicable |

**Global rating**

|  | 1 | 2 | 3 | 4 | N/A |
| --- | --- | --- | --- | --- | --- |
| Overall what is your impression about how this student led the ACP conversation? | Very satisfied | Satisfied | Dissatisfied | Very dissatisfied | Not applicable |

**Written comments**

| Please describe what this learner did particularly well during the ACP conversation |  |
| --- | --- |
| How can this learner further enhance their communication with patients during ACP conversations? |  |
| Additional comments |  |

**Post-Intervention**

***Post-intervention questions to be answered by the preceptor***

Please record your own response to the following questions:

1. On a scale of 1-5 (with 1 = not at all difficult and 5 =extremely difficult), how difficult was it to complete the ACP-CEx form during the resident ACP discussion?

Not at all difficult Extremely difficult

1 2 3 4 5

2. On a scale of 1-5 (with 1 =not at all helpful and 5 =extremely helpful), how helpful was the ACP-CEx in guiding assessment of the resident’s skills in ACP conversations?

Not at all helpful Extremely helpful

1 2 3 4 5

Thank you very much for completing the ACP-CEx assessment. Please take a few minutes after the patient encounter to discuss with the trainee your assessment and the process of ACP. Please return this form to the study coordinator.
