# Supplementary material for: Serious Illness Conversation–Evaluation Exercise: A Novel Assessment Tool for Residents Leading Serious Illness Conversations
Source: Palliat Med Rep. 2020 Nov 24;1(1):280–90. doi: 10.1089/pmr.2020.0086 (PMC8241377; doi:10.1089/pmr.2020.0086)
Supplement: Supplemental data [file Supp_AppS2.docx]

**SIC-Ex for Oncology Trainees Post-Encounter Survey**

Trainee #

Preceptor #

Patient #

EPA/IM stage

Date of discussion

Centre

Please take a moment to complete the questions below after an ACP-CEx session has been completed.

**Feasibility of SIC-Ex (circle one)**

**Please indicate a value from 1 – 4, with 1 indicating the most difficult and 4 indicating the easiest.**

|  | Very difficult |  |  | Very easy |
| --- | --- | --- | --- | --- |
| Finding a patient for ACP discussion | 1 | 2 | 3 | 4 |
| Contacting preceptor to observe discussion | 1 | 2 | 3 | 4 |

**Outcomes of Discussion (circle one)**

**Please answer a value from 1 – 4 with 1 being least and 4 being most satisfactory.**

|  | Completely unsatisfactory | Somewhat unsatisfactory | Somewhat satisfactory | Very satisfactory |
| --- | --- | --- | --- | --- |
| Satisfaction with outcome of discussion | 1 | 2 | 3 | 4 |
| Self-rating of performance of discussion | 1 | 2 | 3 | 4 |
| Self-rating of quality of chart documentation | 1 | 2 | 3 | 4 |

**Self-rating of competence in communication skills**

**Please answer a value from 1 – 4 with 1 being least and 4 being most satisfactory.**

|  | Completely unsatisfactory | Somewhat unsatisfactory | Somewhat satisfactory | Very satisfactory |
| --- | --- | --- | --- | --- |
| Non-verbal communication skills | 1 | 2 | 3 | 4 |
| Verbal communication skills | 1 | 2 | 3 | 4 |

**Self-rating of competence in ACP discussion**

**Please answer a value from 1 – 4 with 1 being least and 4 being most satisfactory.**

**Milestones (CanMEDS Roles):**

**Communication Basics (Professional, Communicator)**

|  | Completely unsatisfactory | Somewhat unsatisfactory | Somewhat satisfactory | Completely satisfactory |
| --- | --- | --- | --- | --- |
| Demonstrated nonverbal empathy  E.g. Sat down, made eye contact | 1 | 2 | 3 | 4 |
| Demonstrated verbal empathy  E.g. Named emotions, understood emotions, stated respect for patient, offered support | 1 | 2 | 3 | 4 |
| Used open-ended questions | 1 | 2 | 3 | 4 |

**Introducing ACP (Professional, Communicator, Health Advocator)**

|  | Completely unsatisfactory | Somewhat unsatisfactory | Somewhat satisfactory | Completely satisfactory |
| --- | --- | --- | --- | --- |
| Introduced ACP as a relevant topic for this patient, e.g. benefit for patient/family, “Hope for the best, prepare for the worst” | 1 | 2 | 3 | 4 |
| Clarified components of ACP previously engaged in | 1 | 2 | 3 | 4 |
| Obtained permission from patient/family to proceed | 1 | 2 | 3 | 4 |

**Learning about the patient (Professional, Communicator, Leader, Scholar)**

|  | Completely unsatisfactory | Somewhat unsatisfactory | Somewhat satisfactory | | Completely satisfactory |
| --- | --- | --- | --- | --- | --- |
| Clarified patient’s understanding of illness (including diagnosis, treatments, prognosis) | 1 | 2 | 3 | 4 | |
| Assessed patient readiness to engage in ACP conversation. E.g. some patients like to know about time, other like to know what to expect, others like to know both, others neither. | 1 | 2 | 3 | 4 | |
| Shared prognosis of current illness with patient, tailored to information preferences | 1 | 2 | 3 | 4 | |
| Inquired about patient’s own values and healthcare goals if medical condition worsens. | 1 | 2 | 3 | 4 | |
| Explored patient’s fears and/or worries with regard to the future of his/her health. | 1 | 2 | 3 | 4 | |
| Explored activities that the patient deems critical to having an acceptable quality of life. | 1 | 2 | 3 | 4 | |
| Explored medical treatments the patient would be willing to go through to gain more time living. | 1 | 2 | 3 | 4 | |
| Explored how much the patient’s family/friends may know about his/her priorities and wishes | 1 | 2 | 3 | | 4 |
| Determined if there were other important friends or family members that needed to be included in future ACP conversations | 1 | 2 | 3 | | 4 |
| Asked who the patient would like as a substitute decision maker | 1 | 2 | 3 | | 4 |

**Planning (Professional, Communicator, Leader, Collaborator)**

|  | Completely unsatisfactory | Somewhat unsatisfactory | Somewhat satisfactory | Completely satisfactory |
| --- | --- | --- | --- | --- |
| Affirmed commitment to continue caring for patient | 1 | 2 | 3 | 4 |
| Acknowledged medical realities | 1 | 2 | 3 | 4 |
| Summarized key goals/priorities | 1 | 2 | 3 | 4 |
| Described treatment options that reflect goals/priorities | 1 | 2 | 3 | 4 |
| Made recommendations about the next steps | 1 | 2 | 3 | 4 |
| Documented conversation | 1 | 2 | 3 | 4 |
| Provided patient with written information pertaining to local ACP policies (e.g. ACP conversation guide) | 1 | 2 | 3 | 4 |

**Educational value of SIC-Ex**

**Please answer a value from 1 – 4 with 1 indicating that the ACP-CEx being most unhelpful and 4 being most helpful in future ACP discussions.**

|  | Very unhelpful | Somewhat unhelpful | Somewhat helpful | Very helpful |
| --- | --- | --- | --- | --- |
| Confidence in conducting future ACP discussions | 1 | 2 | 3 | 4 |
| Competence in conducting future ACP discussions | 1 | 2 | 3 | 4 |
| Overall satisfaction with ACP CEx experience | 1 | 2 | 3 | 4 |

**General attitudes towards the SIC-Ex**

**Please answer yes or no to the following questions:**

The completion of SIC-Ex integrated well with work flow: Yes No

Exercise took an appropriate amount of time: Yes No

Exercise was easy to arrange: Yes No

Faculty observers were accessible: Yes No

Documentation of the discussion was easy: Yes No

The SIC-Ex was an important experience: Yes No

Additional Comments

_________________________________________________________________________________________________________________________________________________________________________________________________________________________________________________________________________________________________________________________________________________________________________________________________________________________________________

Thank you very much for completing the SIC-Ex. Please return this form to the study coordinator.
